# Endocrine and metabolic comorbidities in juvenile-onset systemic lupus erythematosus

**DOI:** 10.3389/fmed.2025.1429337

**Published:** 2025-02-06

**Authors:** Su Jin Park, Moon Bae Ahn, Dae Chul Jeong

**Affiliations:** ^1^Division of Endocrinology, Department of Pediatrics, Seoul St. Mary’s Hospital, College of Medicine, The Catholic University of Korea, Seoul, Republic of Korea; ^2^Division of Rheumatology, Department of Pediatrics, Seoul St. Mary’s Hospital, College of Medicine, The Catholic University of Korea, Seoul, Republic of Korea

**Keywords:** systemic lupus erythematosus, juvenile, endocrine system diseases, metabolic diseases, comorbidities

## Abstract

**Background and aims:**

Juvenile-onset systemic lupus erythematosus (JSLE) is a chronic autoimmune disease affecting individuals under 18, causing multi-system impairment. Patients with JSLE exhibit more severe disease when compared to patients with adult-onset SLE. This study aimed to evaluate the prevalence of endocrine and metabolic comorbidities in patients with JSLE, and analyze the factors associated with each comorbidity.

**Methods:**

Anthropometric, clinical, laboratory data, and the details of glucocorticoids and disease-modifying anti-rheumatic drugs use were collected.

**Results:**

A total of 57 patients with JSLE (48 girls and 9 boys) were included in this study. Endocrine and metabolic comorbidities were observed in 64.9% of the patients. The most prevalent comorbidities were dyslipidemia (40.4%), being overweight or obese (26.3%), subclinical hypothyroidism (24.6%), autoimmune thyroid disease (AITD) (21.1%), and low bone mass (20.9%). The risk of dyslipidemia and AITD increased in patients who were overweight or obese. The risk of being overweight or obese was associated with skin involvement at diagnosis and rheumatoid factor positivity. Younger age at diagnosis and longer duration of glucocorticoid exposure increased the risk of low bone mass. The overall prevalence of endocrine and metabolic comorbidities was associated with short stature at diagnosis, being overweight or obese at follow-up, skin involvement at diagnosis, and rheumatoid factor positivity.

**Conclusion:**

Patients with JSLE have higher burdens of endocrine and metabolic comorbidities and should be routinely monitored. Prevention of obesity may be helpful in lowering the risk of comorbidities.

## 1 Introduction

Systemic lupus erythematosus (SLE) is an autoimmune disease that causes chronic multi-systemic inflammation and tissue damage in various organs, including the skin, joints, heart, serous membranes, blood vessels, nerves, and kidneys (1). Approximately 15–20% of patients with SLE develop the disease before the age of 18 and are therefore diagnosed with juvenile-onset SLE (JSLE) (2). The course of JSLE is often more severe than that of adult SLE, with an increased organ damage, higher frequency of aggressive renal disease, and a higher requirement for steroids and immunosuppressive drugs (3). The overall survival rate of patients with SLE has markedly improved over the past few decades, with up to a 90% 10-year survival observed (4). However, improved survival and a more severe disease course compared with adult SLE indicate that patients with JSLE are at a higher risk of experiencing disease-related morbidities (5).

Several factors may adversely affect the metabolic health of patients with JSLE, including long-standing systemic inflammation, renal involvement, decreased physical activity related to joint pain, limited sunlight exposure, and side effects of treatment. Glucocorticoids (GCs) and hydroxychloroquine (HCQ), an antimalarial drug, are the mainstay long-term treatment in patients with JSLE, often with or without immunosuppressive drugs (6, 7). GCs exert potent anti-inflammatory and immunosuppressive effects and improve the clinical manifestations, laboratory parameters, and disease activity of SLE. Despite its beneficial therapeutic effects, GCs are also associated with well-established adverse effects, and their long-term use may lead to development of metabolic imbalances such as hyperglycemia, type 2 diabetes mellitus (T2DM), dyslipidemia, weight gain, obesity, and metabolic syndrome (8), all of which are well-known risk factors for cardiovascular diseases. Along with disease-specific risk factors such as chronic inflammation, autoantibodies, and cumulative organ damage, SLE is associated with a greater risk of developing cardiovascular diseases and increased mortality (9). Furthermore, considering the early onset of the disease in patients diagnosed with JSLE, long-term GC exposure combined with malnutrition and kidney dysfunction negatively affects growth and puberty, resulting in short stature and pubertal delay (10, 11). Additionally, GCs have detrimental effect on bone mass accrual, leading to low bone density and osteoporosis (12, 13).

Many autoimmune diseases share common pathogenic mechanisms, leading to polyautoimmunity, defined by the presence of multiple autoimmune disease in a single patient (14). SLE is frequently associated with presence of multiple autoantibodies; thus, patients with SLE have a higher possibility of being affected by secondary autoimmune diseases. Although the coexistence of autoimmune endocrinopathies in patients with SLE is mostly described in adults and primarily focuses on autoimmune thyroiditis (15–18), the coexistence of Hashimoto’s thyroiditis (HT) and type 1 diabetes mellitus (T1DM) has been reported in patients with JSLE (14, 19). Autoimmune endocrinopathies may occur at a subclinical level and develop into clinical symptoms later during the disease course (20).

Overall, patients with JSLE have a higher risk of endocrine and metabolic comorbidities, such as being overweight and obese, dyslipidemia, diabetes mellitus, autoimmune thyroid disorders, short stature, delayed puberty, and osteoporosis. This study aimed to evaluate the prevalence of endocrine and metabolic comorbidities in patients with JSLE and analyze the factors associated with each of these endocrine and metabolic abnormalities.

## 2 Materials and methods

### 2.1 Patients

Children and adolescents diagnosed with JSLE at a single institution in Korea between January 2009 and March 2022 were included in this study. Diagnoses were made based on the American College of Rheumatology and Systemic Lupus International Collaborating Clinics classification criteria, by pediatric rheumatologists (21–24). Inclusion criteria were as follows: (1) patients with a symptom onset prior to 18 years of age; (2) patients who had undergone laboratory tests including thyroid function test [free thyroxine (free T4), triiodothyronine (T3), thyroid-stimulating hormone (TSH)], thyroid autoantibodies, and lipid profile [total cholesterol (TC), triglyceride (TG), high-density lipoprotein cholesterol (HDL-C), low-density lipoprotein cholesterol (LDL-C)] at two time points: diagnosis and follow-up; (3) patients who had undergone lipid profile test in a fasting state (at least an 8 h fast), specified as a fasting lipid profile in the medical record; (4) patients who were followed up at least 6 months after the diagnosis.

### 2.2 Clinical and laboratory data of JSLE

Anthropometric, clinical, and laboratory data, including age, height, weight, body mass index, thyroid function, autoantibodies, lipid profile, serologic markers of SLE activity, and the use of GCs and disease-modifying antirheumatic drugs (DMARDs), were collected. All data were collected from each patient at two time points: at diagnosis and at follow-up. Organ involvement was assessed in five categories: skin, joint, hematological and renal, and neuropsychiatric SLE. Skin involvement was categorized based on the presence of malar rash, generalized maculopapular rash, oral ulcers, or photosensitivity. Joint involvement was defined as the presence of swelling or effusion in two or more joints. Hematologic involvement was defined as the presence of one of the following: hemolytic anemia (hemoglobin < 10.0 g/dL with evidence of hemolysis), leukopenia (white blood cell count < 4.0 × 10^9^ /L), and thrombocytopenia (platelet count < 100 × 10^9^ /L). Renal involvement was defined as histological renal damage or proteinuria (>0.5 g) within 24 h. Neuropsychiatric SLE was designated according to the American College of Rheumatology nomenclature (25). Disease activity was assessed using the revised version of the SLE Disease Activity Index (SLEDAI) (26). SLE flare was defined as new or worsening clinical symptoms with escalation of treatment (i.e., new immunosuppressant use, a prednisone increase of 0.5 mg/kg/d, intravenous methylprednisolone, or hospitalization). Serological markers associated with SLE disease activity were also assessed. Low C3 and C4 complement levels and high titers of anti-dsDNA antibodies are considered to reflect high SLE activity (27). The cumulative GC dose (expressed as prednisolone equivalents) was calculated for each patient’s mean body surface area (from diagnosis to follow-up) and presented in g/m^2^. The duration of GC exposure was assessed, excluding periods when GC was discontinued owing to improvement or remission of the disease. The use of HCQ and other DMARDs (azathioprine, methotrexate, mycophenolate mofetil, and cyclosporine) was recorded from diagnosis to follow-up.

### 2.3 Assessment of endocrine and metabolic comorbidities

Endocrine comorbidities were assessed based on anthropometric measurements, clinical manifestations, and laboratory findings. Short stature was defined as a height < 2 standard deviations below the mean for age and sex (28). Growth retardation was defined as growth velocity < 4 cm/year or decreased height *Z*-score > 0.25/year (Δ height *Z*-score < −0.25/year) (11, 29). Growth hormone deficiency (GHD) was defined as a peak growth hormone (GH) level of < 10 ng/mL using two different GH stimulation tests. Diabetes mellitus was defined as HbA1c ≥ 6.5%, with or without islet autoantibodies. Prediabetes was defined as 5.7% ≤ HbA1c ≤ 6.4% (30). Overweight and obesity were defined as BMI ≥ 85th percentile, and obesity as BMI ≥ 95th percentile for age and sex. The presence of dyslipidemia in children and adolescents was defined as at least one of the followings: TC ≥ 200 mg/dL, TG ≥ 130 mg/dL (≥100 mg/dL in children younger than 10 years), LDL-C ≥ 130 mg/dL, HDL-C < 40 mg/dL; in adults, TC ≥ 240 mg/dL, TG ≥ 200 mg/dL, LDL-C ≥ 160 mg/dL, HDL-C < 40 mg/dL. AITD was determined by the presence of anti-thyroid peroxidase antibody (anti-TPO-Ab), anti-thyroglobulin antibody (Tg-Ab), and thyroid-stimulating hormone receptor antibodies (TSH-R-Ab). Subclinical hypothyroidism was defined as elevated TSH levels with normal free T4. In the present study, it was distinguished from the subclinical hypothyroid state of HT by the absence of thyroid autoantibodies.

In 43 of 57 patients, LS BMD *Z*-score was measured by dual-energy X-ray absorptiometry (DXA, Horizon W DXA system^®^, Hologic Inc., Marlborough, MA, USA) and lumbar spine X-rays were collected at last follow-up. The LS BMD *Z*-score was calculated and compared with that of 1,650 healthy age- and sex-matched Korean individuals and adjusted for body size in patients with short stature (31). Low bone mass was defined as LS BMD *Z*-score ≤ −2.0 and osteoporosis was defined as the presence of  ≥ 1 vertebral compression fracture in the absence of high-energy trauma (32).

Primary amenorrhea was defined as the absence of menarche in ≥ 15-year-old girls with developed secondary sexual characteristics or in ≥ 13-year-old girls without signs of pubertal development (33). Secondary amenorrhea was defined as the cessation of regular menses for 3 months or irregular menses for 6 months. Oligomenorrhea was defined as the absence of menstruation for longer than 45-day intervals in adolescents (33). Adrenal insufficiency was determined by low morning cortisol below 3 μg/dL and blunted response to ACTH stimulation test (peak cortisol < 18 μg/dL at 30 or 60 min) (34, 35). For the diagnosis of exogenous Cushing syndrome, medical records were reviewed for cumulative dose and duration of GC exposure, the presence of typical Cushingoid features, growth retardation, and hypertension (36).

### 2.4 Statistical analyses

The outcomes are reported as medians [interquartile range (IQR)] for continuous variables and as numbers and proportions for categorical variables. Anthropometric data, clinical features, disease-related serological markers, thyroid function, thyroid autoantibodies, and lipid profiles were compared between diagnosis and follow-up using Student’s *t*-test or the Wilcoxon rank test for paired samples. McNemar’s test was used for the pairwise analysis of categorical variables between diagnosis and follow-up. Binominal logistic regression was performed to identify the risk factors associated with endocrine and metabolic comorbidities. Univariate analyses were performed for all risk factors, and the risk factors with relevance were selected. Variables identified as significant in univariate analysis were entered into a multiple logistic regression model. To evaluate the factors related to overall endocrine and/or metabolic comorbidities, comparisons of quantitative variables between the groups with or without comorbidities were made using the Student’s *t*-test or the Mann–Whitney U test, and comparisons of frequencies were made by the χ^2^ test or Fisher’s exact test, as appropriate. Statistical calculations were performed using the IBM SPSS Statistics for Windows, version 27.0 (IBM Corp., Armonk, N.Y., United States). Two-tailed *p*-values less than 0.05 were considered significant.

## 3 Results

### 3.1 Anthropometric, clinical, and laboratory data, and pharmacological treatments

A total of 57 patients with JSLE (48 girls and 9 boys) were included in this study. JSLE was diagnosed at a median age of 13.8 (IQR 12.3–16.0) years with a median disease duration of 5.0 (IQR 2.3–7.2) years. Most patients (53/57, 93.0%) were diagnosed with JSLE at pubertal age (boys ≥ 12 and girls ≥ 10 years old, respectively).

The anthropometric, clinical, and laboratory data of the 57 patients at diagnosis and last follow-up are summarized in Table 1. Anthropometric measurements at follow-up were compared with those at diagnosis. There was no significant difference in the *Z*-score of height between diagnosis and follow-up; however, for weight and body mass index (BMI), the *Z*-scores were found to be higher at follow-up (-0.02 vs. 0.27, *p* = 0.043 and -0.10 vs. 0.22, *p* = 0.041, respectively). Hematologic involvement was the most common clinical manifestation observed at diagnosis (66.7%), followed by joint (42.1%) and skin (31.5%) involvements. SLE disease activity was assessed using the SLEDAI and categorized into mild (SLEDAI < 6), moderate (SLEDAI 6–12), and severe (SLEDAI > 12) (26, 37). SLE disease activity decreased from moderate at diagnosis to mild at follow-up (SLEDAI: 9 vs. 2, *p* < 0.001). Serological markers associated with SLE disease activity were also evaluated. Complement levels increased significantly at follow-up (C3: 63 vs. 88.5 mg/dL, *p* < 0.001; C4 7.4 vs. 14.2 mg/dL, *p* < 0.001), whereas anti-dsDNA antibody levels showed no significant differences between the two time points (*p* = 0.181). Eight patients (14.0%) were positive for rheumatoid factor (RF) at follow-up. Eighteen of the 57 patients (31.6%) experienced ≥ 1 SLE flares. There were no significant differences in thyroid function and the proportion of patients with thyroid autoantibodies (anti-TPO-Ab, Tg-Ab, TSH-R-Ab) between the two time points. At diagnosis, DXA and lateral lumbar spine X-ray was performed for 23 of 57 patients (40.4%), with no patients revealing vertebral fractures. At the last follow-up, 43 of 57 patients (75.4%) underwent bone assessment, of whom seven patients (16.3%) had vertebral fractures.

**TABLE 1:** Demographic, clinical, and laboratory data of children and adolescents diagnosed with juvenile-onset systemic lupus erythematosus.

Variables	Diagnosis (*n* = 57)	Follow-up (*n* = 57)	*p*-value
Age, years	13.8 (12.3–16.0)	18.8 (15.8–21.2)	
Diagnosed at prepubertal age^a^, *n.* (%)	4 (7%)	4 (7%)	
Female, *n.* (%)	48 (84.2%)	48 (84.2%)	
Disease duration, years		5.0 (2.3–7.2)	
**Anthropometry**			
Height *Z*-score	0.18 (−0.77–0.88)	0.15 (−0.81–0.78)	0.055
Short stature^b^, *n.* (%)	7 (11.8%)	5 (8.7%)	
Weight *Z*-score	−**0.02 (**−**0.95–0.77)**	**0.27 (**−**0.58–1.01)**	**0.043**
BMI *Z*-score	−**0.10 (**−**0.93–0.68)**	**0.22 (**−**0.74–1.06)**	**0.041**
Overweight/obese^c^, *n.* (%)	11 (19.3%)	15 (26.3%)	0.285
**Clinical profile of SLE**			
Skin involvement	**18 (31.5%)**	**1 (1.7%)**	**<0.001**
Joint involvement	**24 (42.1%)**	**5 (8.7%)**	**<0.001**
Renal involvement	12 (21.0%)	5 (8.7%)	0.059
Neuropsychiatric SLE	2 (3.5%)	2 (3.5%)	
Hematological involvement	**38 (66.7%)**	**16 (28.1%)**	**<0.001**
SLEDAI score	**9 (6–13.5)**	**2** (0–4)	**<0.001**
C3, mg/dL	**63** (35–87)	**88.5 (67.5–97.3)**	**<0.001**
C4, mg/dL	**7.4 (5.0–15.9)**	**14.2 (8.7–18.1)**	**<0.001**
Anti-ds DNA Ab, IU/mL	68.2 (12.2–349.5)	38 (14–78)	
Rheumatoid factor (+)	7 (12.2%)	8 (14.0%)	
Experienced one or more SLE flares, *n.* (%)		18 (31.6%)	
SLICC/ACR DI		1 (0–1)	
**Thyroid function test**			
T3, ng/mL	1.2 (0.9–1.4)	1.2 (0.9–1.4)	
free T4, ng/dL	1.3 (1.1–1.4)	1.1 (1.0–1.3)	
Thyroid-stimulating hormone, μIU/mL	1.9 (1.2–3.7)	3.6 (2.6–7.3)	
Anti-TPO-Ab (+), *n.* (%)	9 (15.8%)	7 (12.3%)	
Tg-Ab (+), *n*. (%)	6 (10.5%)	6 (10.5%)	
TSH-R-Ab (+), *n*. (%)	2 (3.5%)	2 (3.5%)	
**Lipid profile**			
Total cholesterol, mg/dL	142 (122.3–220.8)	165 (134–193)	
Triglycerides, mg/dL	**126** (80–201)	**92** (62–132)	**0.008**
HDL-cholesterol, mg/dL	**37.6** (29–57)	**50** (41–58)	**0.027**
LDL-cholesterol, mg/dL	85.5 (63.5–120.6)	91 (75–115)	
**Bone**			
DXA conducted	**23 (40.3%)**	**43 (75.4%)**	**<0.001**
LS BMD Z-score	−0.1 (−1.1–0.9)	−0.6 (−1.4–0.2)	
Low bone mass^d^	**0/23 (0%)**	**9/43 (20.9%)**	**<0.001**
Vertebral fracture, *n*. (%)	**0/23 (0%)**	**7/43 (16.3%)**	**<0.001**
**Pharmacological treatment**			
Hydroxychloroquine, *n*. (%)		57 (100%)	
Glucocorticoids, *n*. (%)		57 (100%)	
Duration of glucocorticoid exposure, year		3.1 (1.7–5.1)	
Cumulative glucocorticoid dose, g/m^2^		10.3 (5.4–20.6)	
Average daily dose, mg/day		12.7 (8.5–23)	
Azathioprine, *n*. (%)		24 (42.1%)	
Methotrexate *n*. (%)		23 (40.4%)	
Mycophenolate mofetil, *n*. (%)		19 (33.3%)	
Cyclosporine *n*. (%)		10 (17.5%)	
Cyclophosphamide, *n*. (%)		1 (1.8%)	
Biologic DMARDs^e^, *n*. (%)		1 (1.8%)	

*^a^*Prepubertal age defined as male < 12 years, female < 10 years old.

*^b^*Short stature defined as height < 2 standard deviation score (SDS) below the mean for age and sex.

*^c^*Overweight/obese defined as BMI ≥ 85th percentile for age and sex.

*^d^*Low bone mass defined as LS BMD Z-score ≤ −2.0. BMD Z-score was adjusted for body size.

*^e^*Biological DMARDs (disease-modifying anti-rheumatic drugs) include etanercept and adalimumab. All values are expressed as median, IQR (25–75%). BMI, body mass index; SLE, systemic lupus erythematous; SLEDAI, Systemic lupus erythematosus disease activity index; Anti-ds DNA Ab, anti-double stranded DNA antibody; SLICC/ACR DI, Systemic Lupus International Collaborating Clinics/American College of Rheumatology Damage Index; Anti-TPO-Ab, anti-thyroid peroxidase antibody; Tg-Ab, thyroglobulin antibody; TSH-R-Ab, thyroid-stimulating hormone receptor antibody; DXA, dual energy x-ray absorptiometry; LS, lumbar spine; BMD, bone mineral density. Bold values are indicated for *P* < 0.05 (statistically significant).

All the patients were treated with HCQ and GCs. The median daily dose of GCs was 12.7 (IQR 8.5–23) mg, with a median cumulative dose of 10.3 (IQR 5.4–20.6) g/m^2^, over a median duration of GC exposure of 3.1 (IQR 1.7–5.1) years. Other DMARDs or immunosuppressive agents used in the cohort included azathioprine (42%), methotrexate (40%), mycophenolate mofetil (33%), cyclosporine (18%), and cyclophosphamide (1.8%). Rituximab, a biologic DMARD, was used in one patient (1.8%).

### 3.2 Endocrine and metabolic comorbidities in JSLE

**FIGURE 1:**
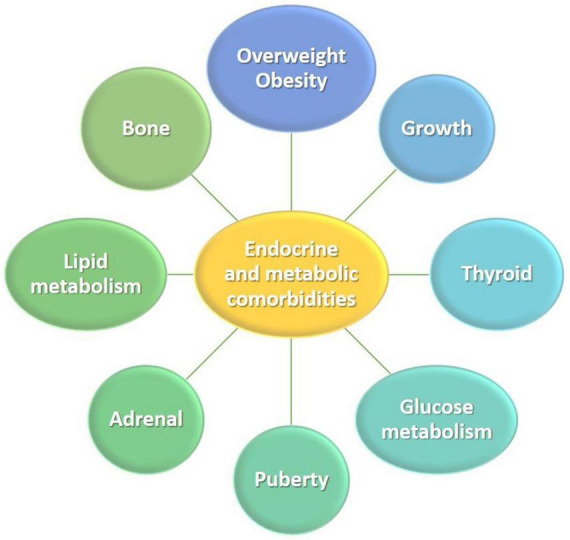
Endocrine and metabolic comorbidities in children and adolescents with juvenile-onset systemic lupus erythematosus.

The prevalence of selected endocrine and metabolic comorbidities, including short stature, GHD, T1DM or T2DM and prediabetes, overweight and obesity, dyslipidemia, autoimmune thyroid disease, subclinical hypothyroidism (non-autoimmune), low bone mass, and osteoporosis, was assessed (Figure 1; Table 2). Notably, for some comorbidities, considering that not all patients routinely underwent clinical or laboratory evaluation for disorders of puberty or the adrenal cortex, the prevalence of these disorders relied on reported cases. Overall, endocrine and metabolic comorbidities were observed in 37 of the 57 patients (64.9%) with JSLE, with the most prevalent comorbidities being dyslipidemia (40.4%, 23/57), being overweight or obese (26.3%, 15/57), subclinical hypothyroidism (24.6%, 14/57), autoimmune thyroid disease (21.1%, 12/57), and low bone mass (20.9%, 9/43).

**TABLE 2:** Endocrine and metabolic comorbidities of children and adolescents diagnosed with juvenile-onset systemic lupus erythematosus.

	No. of patients (%) (*n* = 57)	
**Metabolic disorder**		
Overweight/obesity, *n*. (%)	**15**	**(26.3%)**
Obesity^a^, *n*. (%)	**10**	**(17.5%)**
Dyslipidemia, *n*. (%)	**23**	**(40.4%)**
**Glucose dysregulation**		
Prediabetes, *n*. (%)	**9**	**(15.7%)**
Diabetes mellitus, type 2, *n*. (%)	0	(0%)
Diabetes mellitus, type 1, *n*. (%)	0	(0%)
**Growth**		
Short stature, *n*. (%)	5	(8.8%)
Growth retardation, *n*. (%)	2	(3.5%)
Growth hormone deficiency, *n*. (%)	1	(1.7%)
**Thyroid disorders**		
Autoimmune thyroid disease, *n*. (%)	**12**	**(21.1%)**
Hashimoto’s thyroiditis, *n*. (%)	10	(17.5%)
Hypothyroid, *n*. (%)	4	(7.0%)
Subclinical/Euthyroid, *n*. (%)	5	(8.7%)
Graves’ disease, *n*. (%)	2	(3.5%)
Subclinical hypothyroidism, non-autoimmune, *n*. (%)	**14**	**(24.6%)**
**Bone**		
Dual-energy X-ray absorptiometry conducted, *n*. (%)	43	(75.4%)
Low bone mass, *n*. (%)	**9/ 43**	**(20.9%)**
Osteoporosis, *n*. (%)	**7/ 43**	**(16.3%)**
**Disorders of puberty^b^**		
Primary amenorrhea, *n*. (%)	2	(3.5%)
Secondary amenorrhea, *n*. (%)	3	(5.3%)
Oligomenorrhea, *n*. (%)	3	(5.3%)
Central precocious puberty, *n*. (%)	0	(0%)
**Disorders of the adrenal cortex^b^**		
Iatrogenic Cushing syndrome, *n*. (%)	3	(5.3%)
Glucocorticoid-induced adrenal insufficiency, *n*. (%)	3	(5.3%)
Both Cushing syndrome^c^ and adrenal insufficiency^d^, *n*. (%)	2	(3.5%)
**Overall**		
One or more comorbidities present, *n*. (%)	37	(64.9%)

*^a^*Obesity defined as body mass index ≥ 95th percentile for age and sex.

*^b^*Prevalence of the disorders of puberty and the adrenal cortex relied on reported cases.

*^c^*Iatrogenic Cushing syndrome.

*^d^*Glucocorticoid-induced adrenal insufficiency. Bold values are indicated for *P* < 0.05 (statistically significant).

The anthropometric, clinical, and laboratory data collected at diagnosis and follow-up were used as variables for the analysis of factors related to each endocrine or metabolic comorbidity. The relevant risk factors for each comorbidity with statistical significance are shown in Table 3.

**TABLE 3:** Adjusted risk factors associated with endocrine and metabolic comorbidities in patients with juvenile-onset systemic lupus erythematosus.

Endocrine and metabolic comorbidities	Risk factors	Univariate analysis			Multivariate analysis		
		OR	95% CI	*p*-value	OR	95% CI	*p*-value
Dyslipidemia	Overweight/obese at diagnosis	1.69	0.42–6.76	0.461			
	Overweight/obese at follow-up	**6.51**	1.54–27.41	**0.030**	**5.17**	1.18–22.62	**0.029**
	Renal involvement at diagnosis	2.35	0.61–9.12	0.216			
	Hyperthyrotropinemia^a^	**3.96**	1.17–13.28	**0.027**	3.02	0.84–10.90	0.092
Overweight/obesity	Skin involvement at diagnosis	**3.66**	1.06–12.62	**0.040**	**16.53**	1.51–181.52	**0.022**
	Rheumatoid factor (+)	**6.50**	1.32–31.91	**0.021**	**20.96**	1.42–309.22	**0.027**
	Anti-TPO-Ab (+) at diagnosis	**6.00**	1.17–30.62	**0.031**	6.02	0.77–47.35	0.088
	Tg-Ab (+) at diagnosis	**9.00**	1.33–61.03	**0.024**			
Autoimmune thyroid disease	Overweight/obese at diagnosis	1.54	0.34–7.01	0.575			
	Overweight/obese at follow-up	**6.48**	1.63–25.71	**0.008**	**5.04**	1.18–21.43	**0.029**
	Disease duration	**1.26**	1.02–1.55	**0.032**			
	Rheumatoid factor (+)	**5.13**	1.06–24.87	**0.043**	2.95	0.52–16.70	0.223
Low bone mass	Age of diagnosis	**0.55**	0.36–0.84	**0.006**	**0.55**	0.33–0.91	**0.020**
	Short stature at diagnosis	5.50	0.98–30.80	0.052			
	Duration of glucocorticoid exposure	**1.45**	1.10–1.91	**0.008**	**1.43**	1.01–2.04	**0.045**
	Cumulative glucocorticoid dose	**1.09**	1.02–1.16	**0.007**			
Growth retardation	Age of diagnosis	**0.34**	0.12–0.98	**0.046**	0.40	0.14–1.12	0.082
	Male sex	**13.4**	1.07–168.29	**0.044**	5.53	0.05–555.99	0.467
	Short stature at diagnosis	**19.6**	1.49–256.26	**0.023**			
	Duration of glucocorticoid exposure	**1.56**	1.03–2.37	**0.035**	1.08	0.49–2.39	0.843
	Cumulative glucocorticoid dose	**1.12**	1.02–1.23	**0.018**			

*^a^*Hyperthyrotropinemia is defined as TSH levels above 5 μIU/mL. TSH, thyroid-stimulating hormone; Anti-TPO-Ab, anti-thyroid peroxidase antibody; Tg-Ab, thyroglobulin antibody; CI, confidence interval; OR, odds ratio. Bold values are indicated for *P* < 0.05 (statistically significant).

#### 3.2.1 Dyslipidemia

Dyslipidemia was the most common comorbidity observed in 23 patients (40.4%). When the lipid profiles were compared between the time of diagnosis and follow-up, TG levels were significantly high (126 vs. 92 mg/dL, *p* = 0.008) and HDL-C was significantly low at diagnosis (37.6 vs. 50 mg/dL, *p* = 0.027) (Table 1). At diagnosis, hypertriglyceridemia was associated with severe disease activity [SLEDAI > 12; odds ratio (OR) 3.71, 95% confidence interval (CI), *p* = 0.028] in the logistic regression analysis. At follow-up, TC [*r*(55) = 0.33, *p* = 0.007] and LDL-C [*r*(55) = 0.34, *p* = 0.006] were positively correlated with the average daily GC dose in the Spearman’s rank correlation test.

In a univariate logistic regression analysis, being overweight or obese (BMI ≥ 85th percentile) at follow-up increased the risk of dyslipidemia (OR 6.51, 95%CI, 1.54–27.41, *p* = 0.011), while above-normal BMI at diagnosis did not significantly affect the prevalence of dyslipidemia (Table 3). Hyperthyrotropinemia (TSH > 5 μIU/mL) increased the risk of dyslipidemia (OR 3.96, 95%CI, 1.17–13.28, *p* = 0.027), but the presence of both anti-TPO-Ab or Tg-Ab was not associated with the risk of dyslipidemia. In a multivariate analysis, being overweight or obese at follow-up was the main factor associated with the increased risk of dyslipidemia (OR 5.17, 95%CI, 1.18–22.62, *p* = 0.029). Contrary to predictions, renal involvement at diagnosis, cumulative dose and duration of GCs, and DMARD usage were not significantly associated with dyslipidemia.

#### 3.2.2 Overweight and obesity

As previously noted, in general there was an increase in weight and BMI Z-scores during follow-up compared to those at the time of diagnosis (Table 1). At follow-up, 15 patients (26.3 %) were overweight, of whom 10 (17.5%) were classified as obese (BMI ≥ 95th percentile). The median BMI *Z*-score was 2.17 among overweight and obese patients. Skin involvement at diagnosis (OR 3.66, 95%CI, 1.06–12.62, *p* = 0.040), RF-positivity (OR 6.50, 95%CI, 1.32–31.91, *p* = 0.021), and presence of anti-TPO-Ab (OR 6.00, 95%CI, 1.17–30.62, *p* = 0.031), and Tg-Ab at diagnosis (OR 9.00, 95%CI 1.33–61.03, *p* = 0.024) were the clinical and laboratory factors which affected the prevalence of overweight and obesity in a univariate analysis (Table 3). In a multivariate analysis, skin involvement at diagnosis (OR 16.53, 95%CI, 1.51–181.52, *p* = 0.022) and positive RF (OR 20.96, 95%CI, 1.42–309.22, *p* = 0.027) were associated with increased risk of overweight and obesity. Meanwhile, there was no significant association between being overweight and obese and age at diagnosis, disease duration, SLEDAI score, GC dose, or duration of cumulative GCs.

#### 3.2.3 Thyroid disorders

Thyroid disorders are divided into autoimmune and non-autoimmune thyroid diseases according to the presence of thyroid autoantibodies. Autoimmune thyroid diseases (AITD) include HT and Graves’ disease. Twelve (21.1%) patients had AITD, of whom 10 (17.5 %) had HT and 2 (3.5 %) had Graves’ disease. Non-autoimmune subclinical hypothyroidism was observed in 14 patients (24.6%). Similar to dyslipidemia, being overweight or obese at follow-up was associated with the prevalence of AITD (OR 6.48, 95%CI, 1.63–25.71, *p* = 0.008). Additionally, positive RF (OR 5.13, 95%CI, 1.06–24.87, *p* = 0.043) and disease duration (OR 1.26, 95%CI, 1.02–1.55, *p* = 0.032) were correlated with the prevalence of AITD. In a multivariate analysis, being overweight or obese at follow-up increased the risk of AITD (OR 5.04, 95%CI, 1.18–21.43, *p* = 0.029). There were no identified risk factors related to each subcategory of thyroid disorder: HT, Graves’ disease, and non-autoimmune subclinical hypothyroidism.

#### 3.2.4 Low bone mass and osteoporosis

Among the 43 patients who underwent DXA at follow-up, nine patients (9/43, 20.9%) had low bone mass [lumbar spine (LS) bone mineral density (BMD) *Z*-score < −2.0] after adjustment for body size. Seven patients (7/43, 16.3%) had vertebral compression fractures and were consequently diagnosed with osteoporosis (Table 2). Age of diagnosis (OR 0.55, 95%CI, 0.36–0.84, *p* = 0.006), duration of GC exposure (OR 1.45, 95%CI, 1.10–1.91, *p* = 0.008), and cumulative GC dose (OR 1.09, 95%CI, 1.02–1.16, *p* = 0.007) were the factors associated with low bone mass. Short stature at diagnosis (OR 5.50, 95%CI, 0.98–30.80, *p* = 0.052) showed near significant association with low bone mass (Table 3). Patients diagnosed with JSLE at a younger age had an increased risk of low bone mass. The longer duration of GC exposure and the higher cumulative dose raised the risk of low bone mass. In the multivariate analysis, younger age at diagnosis (OR 0.55, 95%CI, 0.33–0.91, *p* = 0.020) and duration of GC exposure (OR 1.43, 95%CI, 1.01–2.04, *p* = 0.045) remained the main factors which increased the risk of low bone mass. In terms of osteoporosis, age at diagnosis (OR 0.57, 95%CI, 0.37–0.88, *p* = 0.011), short stature at diagnosis (OR 8.63, 95%CI, 1.41–52.83, *p* = 0.020), duration of GC exposure (OR 1.47, 95%CI, 1.09–1.97, *p* = 0.011), and cumulative GC dose (OR 1.09, 95%CI, 1.02–1.16, *p* = 0.010) were the factors associated with osteoporosis. In the multivariate analysis, younger age at diagnosis increased the risk of osteoporosis (OR 0.58, 95%CI, 0.34–0.99, *p* = 0.047), similar to low bone mass.

#### 3.2.5 Glucose dysregulation

Prediabetes was documented in nine patients (15.7%), but none of the patients in the present cohort were diagnosed with T1DM or T2DM. Anthropometric, clinical, and serological factors were not associated with prediabetes risk. High BMI and dose and duration of GCs did not affect the prevalence of prediabetes. Blood glucose level exceeding 105 mg/dL at diagnosis was related to increased likelihood of prediabetes (OR 4.75, 95%CI 1.07–21.03, *p* = 0.040).

#### 3.2.6 Short stature

Seven out of 57 patients had short stature (height standard deviation score < −2) at diagnosis. Of these, five patients remained short, while two of them reached normal height at follow-up (Table 1). One girl patient who had reached normal height was diagnosed JSLE at the age of 12, her height *Z*-score being -2.22. After a year of HCQ and GC treatment, a clinically stable state was achieved. Accordingly, she had changed from daily GCs to alternate-day GCs at the age of 13 years, and had grown 11 cm during the next year, with a final height *Z*-score of -0.45. The other patient who reached a normal height was a boy diagnosed with JSLE at the age of 3 years, when his height *Z*-score was -2.17. His growth rate slowed remarkably to 1.6 cm per year from age 11, and he was diagnosed with GHD when he was 12 years old, his height Z-score being -2.89. He started treatment with recombinant human GH as soon as the coexistence of GHD was noted, and after 2 years of treatment, his height Z-score reached -1.54.

Apart from the patients who had height *Z*-score < −2 upon diagnosis, no patient had short stature in the present study. However, two patients, including the aforementioned boy with GHD, showed growth retardation (growth velocity < 4 cm/year or Δ height *Z*-score < −0.25/year). One of the patients with growth retardation, but not GHD, was a boy diagnosed with JSLE at the age of 8 years with a height *Z*-score of -0.08. Disturbance in growth was noted from the year of diagnosis and progressed until the age of 11 years, with an average growth rate of 2.6 cm/year and height *Z*-score of -1.38. The two patients who showed growth disturbances were both boys and were diagnosed with JSLE at a young age (3 and 8 years, respectively). In a univariate analysis, growth retardation was associated with male sex (OR 13.4, 95%CI, 1.07–168.29, *p* = 0.044), younger age of diagnosis (OR 0.34, 95%CI, 0.12–0.98, *p* = 0.046), and short stature upon diagnosis (OR 19.6, 95%CI, 1.49–256.26, *p* = 0.023). Longer duration (OR 1.56, 95%CI, 1.03–2.37, *p* = 0.035) and higher cumulative dose of GCs (OR 1.12, 95%CI, 1.02–1.23, *p* = 0.018) increased the risk of growth retardation. There were no risk factors from the time of diagnosis identified for short stature. However, endocrine comorbidities including HT (OR 9.69, 95%CI, 1.36–68.34, *p* = 0.023), iatrogenic Cushing syndrome, and GC-induced adrenal insufficiency (OR 16.7, 95%CI, 1.70–162.96, *p* = 0.016) were significantly associated with short stature.

#### 3.2.7 Disorders of puberty

The prevalence of pubertal developmental disorders depends on the number of reported cases. Eight patients (14%) were reported to have disorders of puberty; primary amenorrhea was documented in two patients (3.5%), secondary amenorrhea in three patients (5.3%), and oligomenorrhea in three patients (5.3%). None of the patients experienced central precocious puberty. Menarche did not occur in the two patients with primary amenorrhea until the age of 15, but both patients showed normal development of secondary sexual characteristics for their age. The patients showed no clinical signs of hyperandrogenism (hirsutism, acne, or androgenic alopecia). Laboratory parameters, including luteinizing hormone (LH), follicle-stimulating hormone (FSH), estradiol (E2), testosterone (T), free testosterone (free T), and prolactin (PRL) levels, were within normal ranges. One of these patients experienced menarche at 18 years of age. The other patient had delayed bone age compared to chronological age and a family history of constitutional delay of growth and puberty. As we continuously monitor the occurrence of menarche, she is currently undergoing additional evaluation to assess the presence of a normal uterus using pelvic ultrasonography and progesterone challenge tests.

Six patients with secondary amenorrhea or oligomenorrhea had low-normal-to-normal LH, normal FSH, E2, T, free T, PRL, and thyroid function test results, with adequate pubertal development and bone age for their chronological age. There was no clinical or biochemical evidence of hyperandrogenism. All patients were treated with HCQ and GCs, with a median daily prednisolone equivalent dose of 14.3 mg. Combination treatment with one or more DMARDs, such as methotrexate, azathioprine, and mycophenolate mofetil, was documented in all patients. One patient was administered cyclophosphamide. There were no identified risk factors associated with disorders of puberty, including the dose and duration of GCs or the use of DMARDs.

#### 3.2.8 Disorders of the adrenal gland

The prevalence of adrenal cortex disorders also depends on the number of reported cases. Adrenal dysfunction is classified into two disorders: iatrogenic Cushing syndrome and GC-induced adrenal -sufficiency (AI), both of which are attributed to the long-term administration of GCs. Four patients (7%) had adrenal dysfunction, three patients (5.3%) had iatrogenic Cushing syndrome, three patients had GC-induced AI (5.3%), and two patients had both. The two patients with overlapping disorders manifested centripetal obesity, decreased growth velocity, moon facies, and abdominal striae, and both complained of fatigue and general weakness when GCs tapering was attempted. Laboratory tests for both patients revealed low morning cortisol levels (<3.0 μg/dL) and a blunted response to adrenocorticotropic hormone (ACTH) stimulation test (peak cortisol level < 18 μg/dL). They had used GCs at an average daily dose of 10 mg for 4.1 years and 4.5 mg for 4.2 years, respectively. A girl patient with GC-induced AI alone received an increased GC dose of up to 36.5 mg/day in the last 4 months due to a recent SLE flare. When GC tapering was attempted, she complained of general weakness and was confirmed to have GC-induced AI based on clinical and laboratory findings. One boy patient with iatrogenic Cushing syndrome alone had concomitant GHD, and his Cushingoid features resolved and improved following GH treatment.

Patients diagnosed with JSLE at a younger age had increased risk of iatrogenic Cushing syndrome (OR 0.53, 95%CI, 0.29–0.95, *p* = 0.034). Duration of GC exposure showed marginal association with Cushing syndrome (OR 1.40, 95%CI, 0.96–2.06, *p* = 0.082, data not shown), while cumulative GC dose did not show statistically significant association. Age at diagnosis and duration and dose of GCs did not significantly affect the development of GC-induced AI.

#### 3.2.9 Overall endocrine and metabolic comorbidities

Patients with one or more endocrine and metabolic comorbidities were compared with those without comorbidities. Anthropometric, clinical, biochemical, disease activity, and treatment-related factors were compared between the two groups. Factors significantly associated with the presence of one or more comorbidities are summarized in Table 4. Anthropometric factors including short stature at diagnosis (*p* = 0.045, Fisher’s exact) and being overweight or obese at follow-up (*p* = 0.019, χ^2^) were significantly associated with the prevalence of comorbidities. When analyzing the association between being overweight or obese at follow-up and the presence of comorbidities, two patients, for whom overweight or obesity was the only comorbidity, were reassigned to the non-comorbidity group for accurate analysis. This did not change the model, and being overweight or obese at follow-up remained statistically significant. Among the clinical factors, skin involvement at diagnosis (*p* = 0.048, χ^2^) and RF-positivity (*p* = 0.041, Fisher’s exact) were correlated to the prevalence of comorbidities. Further logistic regression analysis with the extracted variables was performed, but no statistical significance was found in the multivariate analysis. No significant associations were identified between the presence of comorbidities and demographic, biochemical, disease-related, or treatment-related factors.

**TABLE 4:** Factors associated with overall endocrine and metabolic comorbidities in children and adolescents with juvenile-onset systemic lupus erythematosus.

Risk factors	*N*. of patients (%)		*p*-value
	Without comorbidities (*n* = 20)	With comorbidities (*n* = 37)	
Short stature at diagnosis, *n*. (%)	0 (0%)	7 (18.9%)	**0.045**
Overweight/obese at follow-up^a^, *n*. (%)	2/22 (9.1%)	13/35 (37.1%)	**0.019**
Skin involvement at diagnosis	3 (15%)	15 (40.5%)	**0.048**
Rheumatoid factor (+), *n*. (%)	0 (0%)	8 (21.6%)	**0.041**

*^a^*When analyzing the association between overweight/obesity at follow-up and the presence of comorbidities, two patients who had overweight/obesity as the only comorbidity were reassigned from the with comorbidities group to the without comorbidities group, which resulted in the number of patients in the with comorbidities group being 35. Bold values are indicated for *P* < 0.05 (statistically significant).

## 4 Discussion

This study focused on a comprehensive analysis of the prevalence of endocrine and metabolic comorbidities in patients with JSLE and the risk factors associated with individual and overall comorbidities. Endocrine and metabolic comorbidities were observed in 64.9% of patients with JSLE. The most prevalent comorbidity was dyslipidemia, followed by being overweight or obese, subclinical hypothyroidism, autoimmune thyroid disease, low bone mass, and osteoporosis.

Dyslipidemia was the most common comorbidity, which was observed in 40.4% of patients. Cross-sectional studies on patients with JSLE have reported the prevalence of dyslipidemia to be 39–85%, with incidence rates differing according to the definitions of dyslipidemia (39–41). The higher prevalence of dyslipidemia in JSLE compared to the healthy controls results from a complex interaction between chronic inflammation, disease activity, pro-inflammatory cytokines, autoantibodies, decreased renal function, GCs, and the use of other DMARDs (41, 42). Mechanisms such as tumor necrosis factor-alpha (TNF-α)-mediated inhibition of lipoprotein lipase, low lipoprotein activity, and the presence of antibodies to lipoprotein lipase found in patients with SLE have been suggested to contribute to the pathophysiology of dyslipidemia in SLE (41, 43). In our study, the risk of dyslipidemia correlated with being overweight or obese at follow-up and with hyperthyrotropinemia. Hyperthyrotropinemia (elevated TSH) in the present study comprised overt and subclinical hypothyroid states, regardless of the presence of thyroid autoantibodies. Thyroid hormones affect the production and clearance of cholesterol. Recent studies have demonstrated that TSH itself plays a role in lipid metabolism, in addition to the action of thyroid hormones (44). TSH binds to the TSH receptor on hepatocytes and increases the expression and activity of 3-hydroxy-3-methyl glutaryl coenzyme (HMG-CoA) reductase while inhibiting the synthesis of hepatic bile acids, which are necessary for cholesterol catabolism and secretion (44). Therefore, TSH independently induces cholesterol synthesis and blocks its clearance. However, in the multivariate analysis, overweight and obesity remained the only significant risk factors for dyslipidemia. Although the influence of overweight and obesity on dyslipidemia in previous studies on patients with JSLE was not significant, overweight and obesity are widely recognized factors associated with dyslipidemia (45–47). Individuals with obesity typically present with atherogenic dyslipidemia, which is characterized by low HDL-C and elevated TG-rich lipoproteins. This is caused by the relatively reduced insulin-sensitive lipoprotein lipase activity in obesity, which leads to a decrease in HDL-C (48). Previous studies have documented an association between long-term use of GCs and changes in lipid profiles. Chronic use of GCs can increase cholesterol and TG levels *via* increased insulin resistance, increased cholesterol synthesis in the liver, and decreased lipid catabolism (49). A pediatric SLE cohort study demonstrated that TC and LDL-C levels were affected by the daily prednisone dose, and TG levels were affected by changes in disease activity (50). Another study on patients with JSLE reported that TC and LDL-C levels were associated with prednisone dosage and were abnormal only when the disease activity was high (51). In the present study, the prevalence of dyslipidemia was not associated with the cumulative dose or duration of GCs. However, when the average daily GC dose was compared with cholesterol and TG levels, a higher average daily GC dose was correlated with higher TC and LDL-C levels at follow-up. This finding was consistent with previous studies, implying that the daily GC dose is associated with changes in the lipid profile, particularly TC and LDL-C. The only occasionally statistically significant association between GC use and lipid levels found in our study, either when they were evaluated individually or together, may be explained by the interaction between the GCs’ effect on elevating lipid levels, while also controlling the systemic inflammation of SLE, which leads to decreased lipid levels (52).

Overweight and obesity were observed in 26.3% of the patients, which was higher than the prevalence reported in a nationwide survey data (18.6%) in children and adolescents (47). Overweight and obesity were associated with skin involvement at diagnosis, RF positivity, and the presence of anti-TPO-Ab and Tg-Ab. Marzullo et al. (53) had demonstrated a significantly higher rate of anti-TPO-Ab and Tg-Ab positivity in obese patients than in non-obese patients. Another study by Yan et al. reported higher Tg-Ab positivity in obese patients (54). Considering the close association between the prevalence of overweight or obesity, hyperthyrotropinemia, and HT in our study, we assumed that thyroid autoimmunity causes overt or subclinical hypothyroidism, leading to an increased likelihood of obesity. Overt and subclinical hypothyroidism are associated with obesity. Thyroid hormones regulate the basal metabolism rate and thermogenesis, and participate in glucose and lipid metabolism (55). In addition to reduced thyroid hormone, some investigations have shown a positive association between obesity and hyperthyrotropinemia (38, 54, 56, 57). Consequently, overt or subclinical thyroid dysfunction may result in weight gain. Skin involvement and RF-positivity remained the main factors affecting the risk of overweight and obesity in the multivariate analysis. It is noteworthy that there is a link between skin involvement and RF positivity in SLE. RF positivity, observed in approximately 25% of patients with SLE, has been reported to correlate with skin involvement, Sicca syndrome, and less severe nephritis (58–60). In our study, malar rashes were observed in all patients with skin involvement. Malar rash is an acute skin manifestation in SLE and is correlated with disease activity (61). However, the relationship between skin involvement and overweight and obesity was not sufficiently explained by disease activity because there was no significant relationship between SLEDAI score and overweight or obesity in our study. We hypothesized that the marked skin involvement may lead to more aggressive sun avoidance and reduced outdoor physical activity, which may correlate with development of overweight and obesity. However, owing to the lack of sufficient data on the amount of physical activity or physical function assessment, there is insufficient evidence to support this hypothesis. In addition to the significant association between skin involvement and RF positivity, the relationship between obesity and RF positivity was understood as a correlation between obesity and autoimmunity. RF, a biomarker of rheumatoid arthritis (RA), is an autoantibody against the Fc portion of immunoglobulin G (58). The presence of RF can also imply autoimmune activity unrelated to RA, such as organ rejection after transplantation, and can serve as a marker of autoimmunity (62). Children and adolescents with autoimmune rheumatic diseases not only have a high prevalence of being overweight and obese (63), but evidence also suggests that the risk of autoimmune diseases, such as RA and SLE, is increased in obese populations *via* adipokines and pro-inflammatory cytokines (64, 65). Additionally, Giles et al. (66) demonstrated that RF positivity is significantly associated with abdominal obesity. Matsui et al. (67) reported that the concentration of plasma visfatin, a pro-inflammatory adipokine, was significantly correlated with serum RF in patients with RA, suggesting a possible biological link between RF positivity and obesity *via* adipokines. Therefore, we hypothesized that the correlation between RF positivity and overweight and obesity reflects the association between autoimmunity and obesity, suggesting a possible interplay of RF and adipokines, which may contribute to obesity. In our study, no association was found between GC use and the occurrence of overweight and obesity, similar to a large population-based study including 11,288 patients with SLE in Taiwan (68). This suggests that in patients with SLE, a number of other factors affect the occurrence of obesity, beyond GC exposure. These factors may include the abovementioned skin involvement, RF positivity, thyroid autoantibodies, or disease progression, reduced physical activity.

AITD, mainly HT, was observed in 21.1% of the patients. Polyautoimmunity is commonly observed in JSLE patients. In a study of 1,463 patients with JSLE, the most frequent concomitant autoimmune diseases were HT (29%) and antiphospholipid syndrome (29%), followed by autoimmune hepatitis (18%) and T1DM (16%) (19). The prevalence of AITD has been reported to range from 6 to 58% in patients with SLE and JSLE, with varying incidence depending on the definition of AITD (15, 18, 69, 70). The incidence of AITD in our study was associated with overweight and obesity, disease duration, and RF positivity in univariate analysis; overweight and obesity were the main factors in multivariate analysis. The relationship between AITD and obesity is assumed to be mutual. AITD not only affects the prevalence of overweight and obesity by altering the metabolic rate but also vice versa, as overweight and obesity can have an impact on thyroid autoimmunity. During obesity, adipokines such as leptin and adiponectin are released inappropriately. This altered adipokine secretion may promote a shift from a Th2 to a pro-inflammatory Th1 immune response, which is involved in the pathogenesis of autoimmune disorders. Leptin may downregulate the proliferation of Tregs, which are T cells capable of controlling autoimmune reactions. Moreover, adipose tissue secretes a wide range of pro-inflammatory cytokines such as interleukin-6 and TNF-α (53, 71). Consequently, the adipose tissue may participate in the progression of autoimmune inflammatory diseases. Although RF positivity was not the main factor affecting the incidence of AITD, it was a relevant factor for AITD in univariate analysis. This is consistent with previous studies that reported a high prevalence of thyroid autoimmunity in patients with RF-positive RA (72–74). The incidence of thyroid autoantibodies and AITD is significantly higher in RF-positive patients with RA than that in RF-negative patients (72, 73). Another study of 524 patients with SLE also reported more frequent RF positivity in patients with SLE and AITD than in those without AITD (74). The correlation between RF positivity and AITD was interpreted as an extension of the role of RF as a marker for increased autoimmune activity.

Low bone mass was observed in 20.3% of the patients, and 16.3% were diagnosed with osteoporosis. The risk of low bone mass increases with a younger age at diagnosis and longer GC duration. Bone mass steadily increases from birth until the peak bone mass is achieved at approximately 19 and 21 years of age in girls and boys, respectively, and shows the greatest increase from the age of 11 to 13 years in girls and 12 to 14 years in boys (32). Hence, the earlier the patient is diagnosed with JSLE, the more likely they are to be affected by risk factors for secondary osteoporosis, such as chronic inflammation, long-term GCs, decreased physical activity, limited sunlight exposure, and hormonal imbalance, including gonadal insufficiency. Several studies have reported an association between low BMD and GC duration. A study on 702 adults with SLE reported that a longer duration of GC use was an independent risk factor for the time from diagnosis to fracture (75). Michel et al. (76) demonstrated that the number of years of GC use were positively associated with a high fracture rate in a study of 1,110 patients with RA. Steinbuch et al. (77) reported that longer exposure duration significantly increased the risk of hip and vertebral fractures. A recent meta-analysis of osteoporosis in adult patients with SLE also found that the cumulative dose and duration of GCs differed considerably between patients with and without osteoporosis (78). GCs cause bone loss by increasing bone resorption and decreasing bone formation. GCs stimulate osteoclast proliferation by suppressing osteoprotegerin synthesis and upregulating the receptor activator of the nuclear factor kappa-B ligand. They also decrease intestinal calcium absorption and inhibit gonadotropin secretion, resulting in bone resorption. Chronic use of GCs reduces bone formation by inhibiting osteoblast proliferation and stimulating osteoclasts and osteocytes (79).

In the present study, growth retardation was associated with younger age at diagnosis, male sex, short stature at diagnosis, and duration and dose of cumulative GCs. This was in line with a large prospective study on growth and puberty in JSLE, where boys were more growth-affected at a young age at diagnosis, and high cumulative doses of GCs were suggested as major risk factors that adversely affected growth (11). Another study of 97 patients with JSLE reported male sex, short stature at diagnosis, and cumulative GC dose as independent determinants of short adult height (10). GCs hinder linear growth by inhibiting chondrocyte proliferation, hypertrophy, and cartilage matrix synthesis in growth plates (80). GCs can also interfere with GH secretion, downregulate GH receptor expression, and decrease the bioactivity of insulin-like growth factor 1 bioactivity (81). There are reports on the influence of age of onset on outcome in JSLE, and studies have consistently found that a younger age of onset was correlated with a higher risk of organ damage and poorer outcome (82–84). Thus, a younger age at diagnosis indicates that the patient is more likely to experience chronic systemic inflammation and more aggressive treatment from a young age, which may negatively affect their growth. In the present cohort, 22.2% (2/9) of boys and 4.2% (4/48) of girls were diagnosed at a prepubertal age. This difference may imply that more boys had disease onset before the pubertal growth spurt and explain why male sex was associated with an increased risk of growth retardation. The correlation between short stature at diagnosis and increased risk of growth retardation is similar to previous studies that indicated that patients with previous growth failure find it more difficult to catch-up with normal growth rate (11, 85). Short stature is correlated with several comorbidities, including HT and iatrogenic Cushing syndrome. Thyroid hormones are crucial for normal growth and skeletal maturation, and hypothyroidism in children causes growth failure through delayed ossification and mineralization, decline in GHs and insulin-like growth factor 1, and impaired protein synthesis (86). Iatrogenic Cushing syndrome frequently presents with linear growth retardation in children owing to the negative impact of GCs on growth (87).

Adolescents with JSLE often experience delayed puberty, and ovarian dysfunction manifests as menstrual abnormalities, with a prevalence of menstrual abnormalities ranging from 47 to 63% (88–90). Ovarian dysfunction in SLE is hypothesized to be mediated by various factors, including hypothalamic–pituitary–ovarian axis dysfunction, thyroid disorders, and immunosuppressive drugs such as cyclophosphamide and GCs (88). Studies have suggested a correlation between ovarian dysfunction in SLE and a higher damage index, disease duration, cumulative methotrexate, and cyclophosphamide exposure (88, 89, 91–93). We found no significant risk factors associated with menstrual abnormalities in our study, probably because of the small number of self-reported menstrual abnormalities.

The development of iatrogenic Cushing syndrome and GC-induced AI is generally related to the dose and duration of GCs, but a Cushingoid appearance has been observed even at lower doses (68, 94), and evidence to support the dose-dependent risk of GC-induced AI is insufficient in children (95). A younger age at diagnosis was associated with an increased incidence of iatrogenic Cushing syndrome in the present study. It was assumed that younger age at diagnosis, which correlated with a greater risk of organ damage, led to a more prolonged supraphysiological dose of GCs, resulting in iatrogenic Cushing syndrome. Studies have reported the prevalence of GC-induced AI to be 12.8% in adults with SLE and 32% in children with rheumatic diseases (96, 97). The lower prevalence of adrenal insufficiency in the present study was presumed to be due to the inclusion of documented cases only.

As previously mentioned, polyimmunity is common in SLE, and T1DM has been observed in 5–20% of patients with JSLE in prior studies (19, 98, 99). Several studies have shown that insulin resistance is more common in patients with SLE, increasing the risk of T2DM. The prevalence of T2DM is 3–25%, varying depending on the diagnostic criteria for diabetes (100, 101). Although no patients were diagnosed with DM in our study, prediabetes was observed in 15.7% of patients, presumably due to the higher prevalence of insulin resistance in JSLE, the chronic pro-inflammatory state, and the hyperglycemic effect of GCs (101).

The overall prevalence of endocrine and metabolic comorbidities was associated with four factors: short stature at diagnosis, overweight or obesity at follow-up, skin involvement at diagnosis, and RF positivity. RF positivity linked to skin involvement in SLE may aid pediatric endocrinologists in identifying individuals at risk for endocrine and metabolic comorbidities. Interestingly, RF positivity in SLE has been suggested to play a protective role against lupus nephritis (58–60). Hence, in the context of organ damage, it can be considered an indicator of a benign disease. However, the present study focused on the prevalence of endocrine and metabolic comorbidities rather than on cumulative organ damage. RF positivity in JSLE may be interpreted as an indicator reflecting autoimmunity and metabolic derangements, namely overweight and obesity in the present study, rather than as a pathogenic marker as in RA. Height represents the genetic, biological, and environmental factors that affect individuals during childhood, such as nutrition, hormonal balance, and chronic diseases. In view of short stature as a reflection of previous exposures from the early years of life, it can be inferred as a risk factor for endocrine and metabolic comorbidities *via* suboptimal regulation of nutrition, hormonal balance, and chronic diseases. Lastly, overweight and obesity at follow-up were the only modifiable risk factors among those significantly associated with comorbidities, indicating that the prevention of overweight and obesity may be helpful in lowering the risk of endocrine and metabolic comorbidities.

The current study had some limitations owing to its retrospective design. First, the total number of patients with JSLE who underwent thyroid function tests and lipid profiling at diagnosis was small. The results were derived from a single-center study in Korea, which restricts their applicability to broader JSLE populations with various ethnic or geographic backgrounds. Second, comorbidities, such as disorders of puberty and adrenal dysfunction, were screened only if the patients were symptomatic or clinically suspected; consequently, the actual prevalence of these two disorders may have been underestimated, affecting the reliability of the associations found. Third, not all patients had data on mid-parental height and bone age; hence, we used absolute height to define short stature, and the effects of genetic predisposition and pubertal development were mitigated. Fourth, although the SLEDAI score at diagnosis and follow-up, along with the number of flares, have been investigated, they may not fully capture the cumulative disease activity of SLE. Lastly, the present findings provide valuable insights into the associations between the comorbidities and the risk factors, but due to the cross-sectional design of the study, there are limitations in establishing the definite causal relationships between the comorbidities and the risk factors. However, the strength of this study is that we performed a comprehensive analysis of the prevalence of endocrine and metabolic comorbidities in patients with JSLE and the effects of anthropometric, clinical, laboratory, and treatment-related variables on the occurrence of comorbidities.

In conclusion, children and adolescents with JSLE have a high prevalence of endocrine and metabolic comorbidities. Clinicians should cautiously monitor for the potential coexistence of endocrine and metabolic abnormalities in patients with JSLE, particularly dyslipidemia, overweight and obesity, autoimmune thyroid diseases, and low bone mass. Managing modifiable factors such as overweight and obesity may be helpful in lowering the risk of endocrine and metabolic comorbidities. More longitudinal studies with patients with JSLE are required to establish the causal relationship between the comorbidities and the risk factors, and elucidate the impact of interventions such as weight loss on the comorbidities.

## Data Availability

The original contributions presented in the study are included in the article/supplementary material, further inquiries can be directed to the corresponding author.
